# Efficacy and safety of lavender essential oil (Silexan) capsules among patients suffering from anxiety disorders: A network meta-analysis

**DOI:** 10.1038/s41598-019-54529-9

**Published:** 2019-12-02

**Authors:** Wuan Shuen Yap, Anton V. Dolzhenko, Zahraa Jalal, Muhammad Abdul Hadi, Tahir Mehmood Khan

**Affiliations:** 1 School of Pharmacy, Monash University Malaysia, Jalan Lagoon Selatan, 47500 Bandar Sunway, Selangor Darul Ehsan Malaysia; 2 School of Pharmacy, Curtin Health Innovation Research Institute, Faculty of Health Sciences, Curtin University, GPO Box U1987, Perth, Western Australia 6845 Australia; 3 School of Pharmacy, College of Medical and Dental Sciences, Institute of Clinical Sciences, University of Birmingham, Birmingham, B15 2TT England, UK; 4 The Institute of Pharmaceutical Sciences (IPS), University of Veterinary & Animal Sciences (UVAS), Outfall road, Lahore, Pakistan

**Keywords:** Drug therapy, Drug therapy, Drug therapy, Sleep disorders, Sleep disorders

## Abstract

A systematic review and network-meta analysis (NMA) were performed to estimate significance of the anxiolytic effect of lavender essential oil taken as silexan capsules versus other comparators (i.e., placebo/paroxetine/lorazepam). The outcome of interest was Hamilton Anxiety Scale (HAMA). Weighted mean differences (WMD) were calculated to estimate the treatment effect at the confidence interval of 95%. League tables were generated using treatment effect, for all pairwise comparisons, where WMD < 0 favors the column-defining treatment. Five studies were identified with a total of 524 participants receiving treatment with silexan 80 mg and 121 participants taking silexan 160 mg. The NMA results indicated that consumption of silexan 160 mg resulted in higher decline of HAMA score [WMD −1.14 (−1.10, 3.39)] in comparison to silexan 80 mg, placebo [−2.20 (−4.64, 0.24)] and paroxetine [−1.24 (−5.34, 2.85)]. The effect of silexan 80 mg was observed to be same as that of paroxetine. Overall, silexan 160 mg was noticed to be a more efficient treatment giving significant decline in HAMA score across other comparators. However, no improvements in HAMA score was observed for the group receiving lorazepam 0.5 mg when compared to silexan 160 mg, silexan 80 mg, paroxetine 20 mg, and placebo.

## Introduction

Among psychiatric disorders, anxiety disorders are more frequent than others^1^. The global prevalence of anxiety disorders based on the data from 87 studies in 44 countries around the world was estimated to be 7.3%^2^. The frequency of anxiety in the population differs greatly between countries. The lifetime prevalence of anxiety disorders in United States was reported to be 28.8%^3^, 14.5% in Europe^4^ and 20% in Australia^5^. Anxiety disorders have a huge economic impact on society affecting mainly working-age population. Thus, the prevalence of anxiety disorders in United States for the 18–64 years old population reaches 33.7%^6^. Anxiety often manifests as a symptom of other psychiatric disorders and also frequently precedes their onset^7,8^. Anxiety disorders are also more comorbid than other mental disorders^9^. Their high comorbidity extended to other psychiatric disorders as well as physical illnesses.

Due to chronic nature of anxiety disorders, patients suffer from them for a long time, sometimes decades. However, therapeutic interventions, such as pharmacotherapy and psychotherapy and their combination, are usually beneficial and improve patient’s quality of life often resulting in complete recovery^10,11^. The meta-analysis estimating efficacy of treatments for anxiety disorders suggested that positive effects of pharmacotherapy exceeded those of psychotherapy^1^. Therefore, pharmacotherapy is often critically important in managing patients with anxiety disorders. However, the chronic character of anxiety disorders and requirements of the long-term treatment set very high safety and compliance standards for the medications and bring phytotherapy as a treatment option^12^.

Phytotherapy has been gaining popularity in the treatment of anxiety^13–17^ with many GABA-modulating medicines of herbal origin undergoing preclinical and clinical investigations^18^. Particular attention has been paid to anxiolytic-like effects of essential oils, among which a lavender essential oil demonstrated the best pharmacological profile^19–21^.

Lavender (*Lavandula angustifolia* Miller or *Lavandula officinalis* Chaix) has a long history of traditional use and its essential oil was found to possess a wide range of biological effects^22,23^. Evidence of the effectiveness of the lavender essential oil in the pharmacotherapy of mental disorders led to the development of Silexan, which is a standardized essential oil of *L. angustifolia* flowers prepared by steam distillation^24^. Silexan is approved in Germany for the treatment of restlessness related to anxiety and marketed as LASEA^®^^25^.

Silexan was found to contain 36.8% of linalool and 34.2% of linalyl acetate^26^. Other components of the lavender essential oil present in substantial quantities include monoterpene alcohol lavandulol, its ester lavandulyl acetate, and bicyclic monoterpenoids borneol, eucalyptol (1,8-cineole) and camphor^27,28^. Linalool also demonstrated anxiolytic properties in several animal models^29,30^ and possessed stress-relieving effect in humans under the experimental stress^31^. Studies on molecular mechanisms of pharmacological effects of lavender essential oil revealed that its effect on CNS could be attributed to the inhibition of voltage dependent calcium channels^26^. Unlike some other monoterpenes found in the lavender essential oil, linalool and linalyl acetate significantly inhibited voltage dependent calcium channels^26^. In another study, lavender essential oil was demonstrated to possess affinity to the NMDA receptor and SERT^28^. Binding to NMDA receptor was also observed for major constituents of lavender essential oil linalyl acetate and linalool, while only linalool demonstrated significant binding to SERT^28^. Further results of the clinical, randomized, placebo-controlled, double-blind, cross-over study on healthy men using positron emission tomography technology suggested that the anxiolytic effect of lavender essential oil (given as Silexan) could be attributed to the serotonergic system changes, particularly at the 5-HT_1A_ receptor level^32^.

To date, two reviews have been reported in the literature evaluating the efficacy of Silexan in anxiety-disorders^33,34^. Review by Kasper^33^ in 2013 was not a systematic review, liable to bias, and the author did not combine results of individual studies using meta-analysis. The systematic review and meta-analysis by Möller *et al*.^34^ estimated the efficacy of Silexan in sub-threshold anxiety disorders and reported significant reduction in anxiety as measured by HAMA scale. The present systematic review further builds on our existing knowledge on the efficacy of Silexan in the management of anxiety disorders by comparing the efficacy of different strengths of lavender oil using a network meta-analysis technique. This is a unique technique and is considered superior to meta-analysis when direct multiple comparisons between the intervention and control group do not exist in the literature^35^. In addition, our systematic review has evaluated safety of lavender oil as well.

## Methods

A systematic review was performed to identify potential research papers across 8 databases from inception till December 14, 2017. The NMA was performed, in accordance with the recommendations made by the Preferred Reporting Items for Systematic Reviews and Meta‐Analyses (PRISMA)^36^, to estimate the overall effectiveness of lavender versus placebo and other medicinal products in anxiety disorders and its adverse effects.

### Population intervention comparator and outcomes

- Population of interest: Patients with anxiety disorder.
- Intervention: Lavender given orally (capsules).
- Comparators: Placebo or any other medicinal product.
- Outcome: Change in Hamilton Anxiety Scale (HAMA) total score of the patients.

### Search strategies

Eight electronic databases including Embase via Ovid, MEDLINE via Ovid, Cochrane, PubMed, AMED, PsycINFO, SCOPUS and EBSCOHost using the following search terms: lavender OR lavandula OR alhucema OR aspic OR lavandin OR lavender essential oil OR lavender oil OR lavandin oil OR aspic oil OR lavender extract OR Silexan OR Lasea OR Kalms Lavender One-A-Day OR CalmAid OR MS 1265 OR linalool OR linalyl acetate AND anxiety.

**Figure 1:**
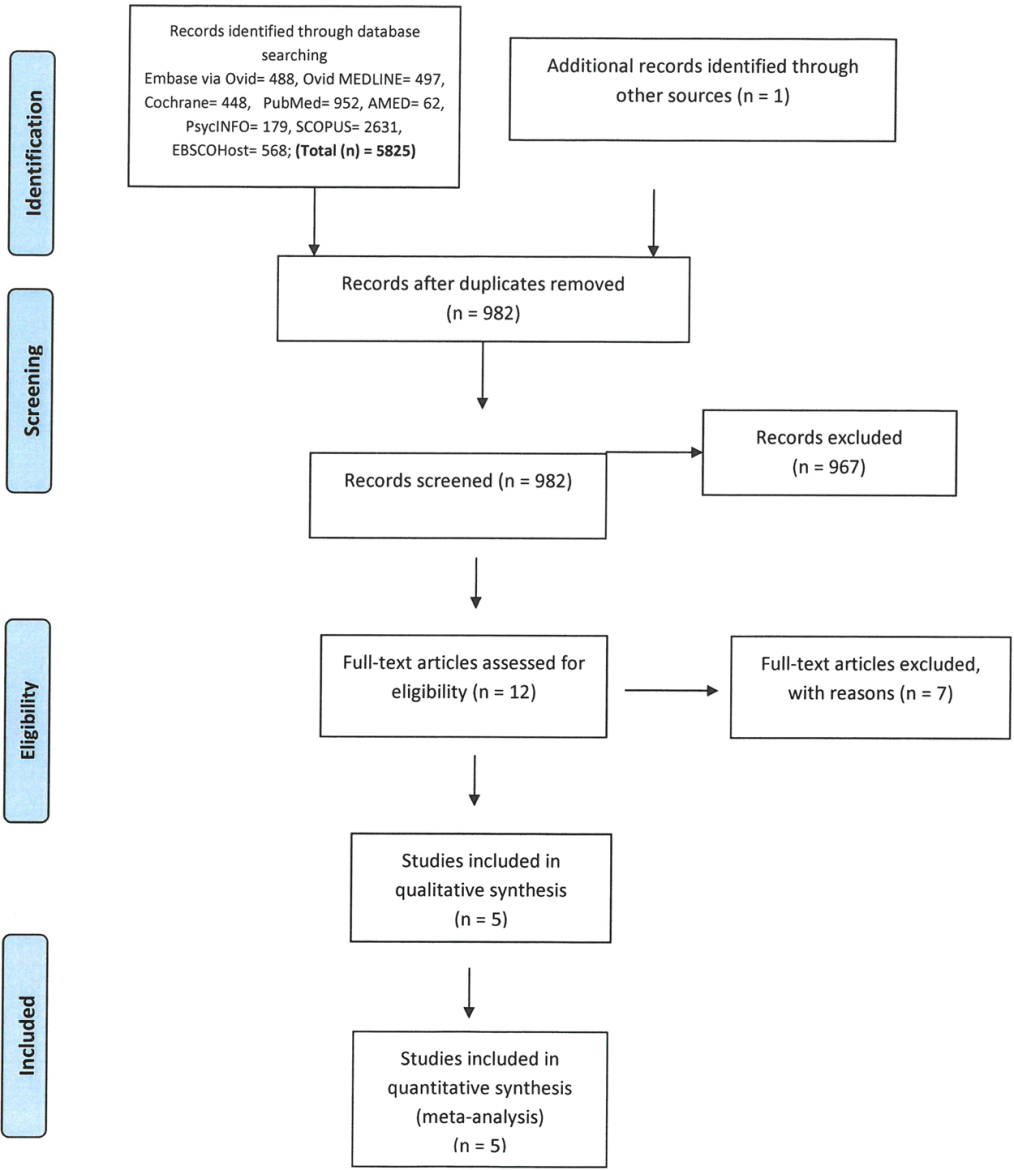
PRISMA flow diagram.

All the relevant papers were identified and imported to an EndNote file to create a combined library. Duplicate records were removed, where appropriate. Title and abstracts were screened by two authors independently and disagreements were resolved through discussions. Full-texts were downloaded and considered for inclusion based on the inclusion/exclusion criteria described below. Also, we manually reviewed the references of the included studies to identify other relevant studies. Additional details about the studies identified are shown in Fig. 1.

### Study selection

The following criteria were used to further align the search with the specific outcomes of interest.

#### Inclusion criteria

- All English language studies from inception till December 14, 2017 were considered eligible for inclusion in this systematic review.
- Only human experimental studies, which are clinical trials comparing the effect of lavender (as a tea, powder or capsule) were considered for potential inclusion in order to assess causal assertions. Comparators considered appropriate were: a placebo, reference/control group, or any other active regimen, which was compared with the lavender formulation given orally.
- The main outcome of interest is the change in Hamilton Anxiety Scale (HAMA) total score of the patients.

#### Exclusion criteria

- Systematic reviews, all observational studies, letter to the editors, case reports, case series, personal opinion, qualitative studies, and reviews/communications focusing on individual experience of the use of lavender were excluded.
- In addition, any data that are not published, reports, and thesis were excluded.
- Experimental studies on animals and studies using lavender for aromatherapy were excluded.

### Outcome of interest

- Primary outcomes: Change in Hamilton Anxiety Scale (HAMA) total score of the patients.
- Secondary outcome: Safety and tolerability of lavender preparations as a medicine.

### Data extraction

Data were extracted independently by two review authors (WSY, TMK) and in the case there was a conflict, a third independent assessor AVD assisted in revalidating the data and resolving the conflict. A structured, pilot-tested data collection form was designed to collect data from individual studies. In addition to the data related to the outcomes of interest, data on the number of authors, year of publication, study design, setting and country where the study took place, sample size, patients’ mean age and gender, and inclusion criteria and exclusion criteria were also extracted. The results for the outcome measures included in this review were summarized as mean and SD difference from baseline to endpoint in both the intervention and control groups. When not reported, the mean and SD difference were calculated provided sufficient data were available.

### Data analysis

The risk of bias was assessed individually for each RCT included in this review using Cochrane risk of bias assessment tool^37^. Two authors (MAH, ZJ) assessed risk of bias independently and disagreements were resolved through discussion.

The network meta-analysis (NMA) was performed using STATA version 14®. Random effects model was used and weighted mean differences with 95% confidence interval (CI) were calculated for all continuous outcome measures to compare the effectiveness of the intervention. P-value less than 0.05 was considered statistically significant. Furthermore, in order to generate forest plots for NMA, respective pairwise comparisons for the treatment effect were carried. League tables were created using treatment effect calculated based on weighted mean difference (MD; 95% CI) for all pairwise comparisons. A league table, a square matrix consisting of all pairwise comparison within a meta-analysis, empowers researcher/reader to directly compare the direction and magnitude of treatment effect, encouraging easier interpretation of results^35^. A negative (−) MD indicate decline in the score for the HAMA and SAS scales.

## Results

### Included studies

Of the 982 studies screened, five randomized control trials were included in this review (Fig. 1).

### Characteristics of the studies and type of interventions

To assess the effect of lavender in treating anxiety, interventions included in the 5 studies were placebo, 80 mg/d silexan, 160 mg/d silexan, 0.5 mg/d lorazepam, and 20 mg/d paroxetine. The study by Woelk & Schalfke^38^ had a treatment phase of 6 weeks whereas the other 4 studies by Kasper *et al*.^39–42^ had a treatment phase of 10 weeks. All these studies were conducted in Germany. The primary outcome in each study was the change in total score of HAMA. Safety and tolerability of the interventions were assessed by the reporting of adverse events that occurred over the course of the study. Details on study characteristics are available in Table 1.

**Table 1:** Study characteristics.

Author	Recruitment site	Design	Study period		Sample size (Intervention/Control)		Intervention(s)	Comparison(s)	Measurement tools		
			Breakdown	Total (for each participant)	Full analysis set	Per-protocol			Outcome	Compliance	Safety and tolerability
Woelk & Schlafke (2009)^35^ Germany	General practitioner’s clinics	Multi-centre, double-blind, randomised phase III study	1 week screening phase, 6 weeks treatment phase, 2 weeks discontinuation phase.	9 weeks	40/37	36/33	80 mg Silexan capsule + Lorazepam placebo once daily during 6-week treatment phase, and on day 43,45,47,50 and 53 during the discontinuation phase.	0.5 mg Lorazepam capsule + Silexan placebo once daily during 6-week treatment phase, and on day 43,45,47,50 and 53 during the discontinuation phase.	Hamilton Anxiety Rating Scale (HAMA total score)	Method was not reported	Adverse events (AE) reported, physical and ECG examinations, vital signs, routine laboratory measurements
Kasper *et al*. (2015)^38^ Germany	17 general and psychiatric practices	Randomized, double-blind trial	Single-blind screening and wash-out phase of 3–7 days, followed by 10 weeks treatment phase. Post-baseline efficacy and safety assessments performed every 2 weeks	11 weeks	86/84	73/65	80 mg Silexan capsule once daily	Placebo capsules with 1/1000 the amount of lavender oil in Silexan capsules (to match the smell of Silexan capsules) once daily	Hamilton Anxiety Rating Scale (HAMA total score)	Compared the number of unused capsules returned by the patients to the expected number assuming a fully protocol compliant intake	Adverse events (AE) reported spontaneously, physical and ECG examinations, vital signs, routine laboratory measurements
Kasper, *et al*. (2016)^39^ Germany	35 psychiatric practices	Double-blind, randomized, placebo-controlled, parallel-group multicentre trial	Screening period of 3–7 days, followed by 10 weeks treatment phase. Efficacy and safety assessments performed at 1 and 2 weeks ± 2 days, as well as at 4, 7 and 10 weeks ± 7 days after baseline	11 weeks	159/156	141/128	80 mg Silexan capsule once daily	Placebo capsules with 1/1000 the amount of lavender oil in Silexan capsules (to match the smell of Silexan capsules) once daily	Hamilton Anxiety Rating Scale (HAMA total score)	Assessed by counting of returned medication	Adverse events (AE) reported, physical and ECG examinations, vital signs, routine laboratory measurements
Kasper, *et al*. (2014)^37^ Germany	57 psychiatric and general practices	Randomized, double-blind, double-dummy, multi-centre trial with four parallel groups	Screening and wash-out phase of 3–7 days, followed by 10 weeks treatment phase. Efficacy and safety assessments performed after 2, 4, 6, 8 and 10 weeks. Treatment phase is followed by 1 week down-titration phase for paroxetine withdrawal	12 weeks	121 (Silexan 160 mg/d)/13 5 (Silexan 80 mg/d)/132 (paroxetine)/135 (placebo)	103 (Silexan 160 mg/d)/119 (Silexan 80 mg/d)/114 (paroxetine)/114 (placebo)	160 mg Silexan capsule once daily; 80 mg Silexan capsule once daily.	20 mg Paroxetine capsule; placebo capsule.	Hamilton Anxiety Rating Scale (HAMA total score)	Assessed by medication counting	Adverse events (AE) reported spontaneously, physical examinations, routine laboratory measurements. Withdrawal symptoms ascertained by administering the Physician Withdrawal Checklist (PWC-20) at the end of treatment phase and down-titration phase.
Kasper, *et al*. (2010)^36^ Germany	27 general and psychiatric primary care centres	Randomized, double-blind, placebo controlled, multi-centre trial	Single-blind placebo screening and wash-out phase of 3–7 days, followed by 10 weeks treatment phase. Post-baseline efficacy and safety assessments performed every 2 weeks	12 weeks	104/108	87/90	80 mg Silexan capsule daily	Placebo capsules with 1/1000 the amount of lavender oil in Silexan capsules (to match the smell of Silexan capsules) once daily	Hamilton Anxiety Rating Scale (HAMA total score)	Method was not reported	Adverse events (AE) reported, physical and ECG examinations, vital signs, routine laboratory measurements

Although the recruitment criteria for all 5 studies included participants of both genders and of any ethnicity, less than 31% of participants in each study were male and almost all were Caucasian. Woelk & Schlafke^38^ conducted a multi-center, double-blind, randomised phase III study assessing the comparative effect of 80 mg silexan daily versus 0.5 mg lorazepam daily on a sample size of 78 participants with 23.4% being male. In this study, 40 participants received silexan 80 mg. Kasper *et al*.^39^ investigated the effect of 80 mg silexan daily against placebo in a randomized, double-blind, multi-center trial with 20.2% of its 228 participants being male. In this study, 104 participants received treatment with silexan 80 mg.

Following that, Kasper *et al*.^40^ conducted a randomized, double-blind, double-dummy, multi-centre trial with four parallel groups receiving 160 mg silexan daily (121 patients), 80 mg silexan daily (135 patients), 20 mg paroxetine daily, and placebo with 26.4% of male in the sample size of 616 participants. Subsequently, Kasper *et al*. in 2015^41^ investigated the effect of 80 mg silexan daily and used placebo for the reference group. The study design was a randomized, double-blind trial with 28.2% of the study’s 179 participants being male^38^. In this study, 86 participants were assigned to the silexan 80 mg group. The following year, Kasper *et al*. published another paper to again investigate the effect of 80 mg silexan daily compared to placebo^42^. In this double-blind, randomized, parallel-group multi-centre trial, the sample size was 362 participants and 30.8% of them were male. The silexan 80 mg group consisted of 159 participants^42^. Further details on participant characteristics are shown in Table 2.

**Table 2:** Participant Characteristics.

Author	Respondents	Sample size			Male; n (%) Full Analysis Set	Age (in years)			Ethnic composition n(%)
		Screened	Randomized	Completed study		Range	Median	Mean ± SD	
Woelk & Schlafke (2009)^35^ Germany	Patients (18–65 years old) with a primary diagnosis of generalised anxiety disorder (GAD) according to DSM-IV (300.02) and outpatient treatment by a general practitioner.	78	77	74	18 (23.4)	21–65	N/A	N/A	N/A
Kasper *et al*. (2015)^38^ Germany	Male and female patients of any ethnicity (18 and 65 years old) with restlessness and agitation according to the criteria of ICD-10 diagnostic category R45.1, Hamilton Anxiety Rating Scale (HAMA) of at least 18 points, with minimum scores of 2 points for HAMA items “Tension” and “Insomnia” and Pittsburgh Sleep Quality Index (PSQI) of at least 6 points.	179	170	148	48 (28.2)	Silexan 22–67; Placebo 21–67	Silexan 49; Placebo 48	N/A	Caucasian 169 (99.4); Asian 1 (0.6)
Kasper, *et al*. (2016)^39^ Germany	Male and female patients of any ethnicity (18 and 65 years old) with mixed anxiety and depressive disorder (MADD) according to the criteria of ICD-10 criteria and Hamilton Anxiety Rating Scale (HAMA) of at least 18 points, with minimum scores of 2 points for HAMA items “Anxious mood” and “Depressed mood”.	362	318	290	97 (30.8)	N/A	N/A	Silexan 47.7 ± 12.6; Placebo 47.9 ± 12.6	Caucasian 316 (99.4); Asian 1 (0.3); African 1 (0.3)
Kasper, *et al*. (2014)^37^ Germany	Male and female patients of any ethnicity (18 and 65 years old) with generalized anxiety disorder (GAD) according to DSM-IV-TR and ICD-10 (F41.1), Hamilton Anxiety Rating Scale (HAMA) of at least 18 points, with minimum scores of 2 points for HAMA items “Anxious mood” and “Tension”, HAMA subscore for psychic anxiety of less than 21 points and Covi Anxiety Scale total score of at least 9 points	616	539	450	138 (26.4)	N/A	N/A	Silexan 160 mg/d 47.1 ± 11.8; Silexan 80 mg/d 45.7 ± 11.5; Paroxetine 45.8 ± 12.4; Placebo 44.6 ± 12.3	Caucasian 537 (99.6); Others (0.4)
Kasper, *et al*. (2010)^36^ Germany	Male and female outpatients of any ethnicity (18 and 65 years old) suffering from an anxiety disorder according to DSM-IV or ICD-10 F41.9, Hamilton Anxiety Rating Scale (HAMA) of at least 18 points, with minimum scores of 2 points for HAMA items “Anxious mood” and “Insomnia” and Pittsburgh Sleep Quality Index (PSQI) of at least 5 points.	228	216	187	43 (20.2)	N/A	N/A	Silexan 45.6 ± 11.4; Placebo 46.6 ± 11.3	N/A

### Risk of bias

**Figure 2:**
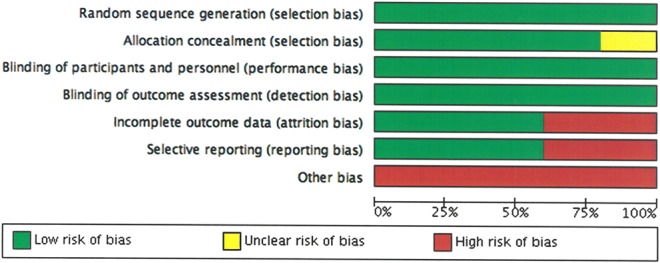
Summary of risk of bias.

**Figure 3:**
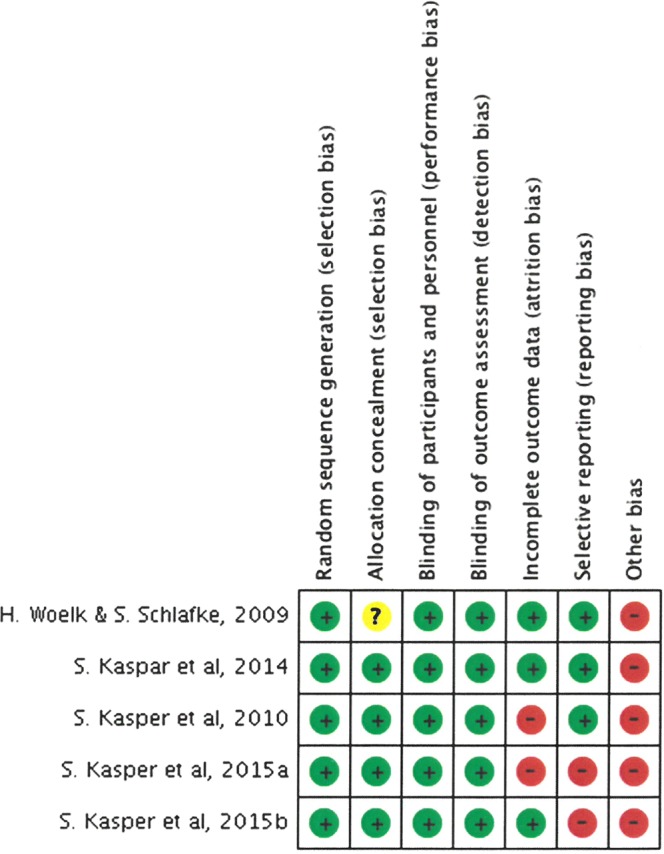
Risk of bias among studies.

In general, the overall risk of bias was low for most of the domains for the included studies (Figs. 2 and 3). In particular, risk was low for selection bias, performance bias and detection bias for all the studies. One of the studies has unclear risk of bias for allocation concealment (Figs. 2 and 3). In other biases, studies were deemed to be high risk of bias as they were funded by the manufacturer.

### Effect on HAMA score: Primary outcome

**Figure 4:**
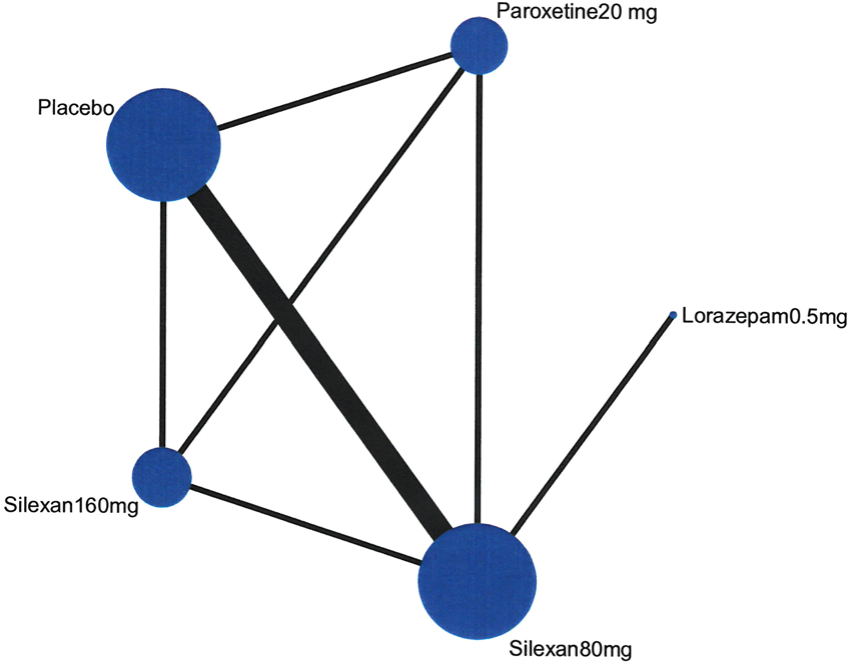
Network Plot.

To estimate the effect of all interventions on HAMA, five studies were included and a NMA was performed to compare and contrast the effect on the HAMA scale. The network plots demonstrate the organization of all available evidences. The width of the lines represents the number of trials and the size of node represents the sample size (Fig. 4).

### Overall analysis

#### Outcome of interventions versus placebo at HAMA scale

Multivariate analysis was performed to estimate the effect of all the active treatments silexan 160 mg, silexan 80 mg, lorazepam 0.5 mg, and paroxetine 20 mg versus placebo as the reference arm. Results have revealed that effect of placebo was not superior in comparison to silexan. Overall, silexan 160 mg −4.963 [−7.167–−2.759], p ≤ 0.001, I^2^ = 0.00%, Tau^2^ = 0), and silexan 80 mg −3.820 [−5.261–−2.380], p ≤ 0.001, I^2^ = 0.00%, Tau^2^ = 0), were noticed to have a significantly higher decline in HAMA score in comparison to placebo. Detailed comparison of all treatment with reference arm is shown in Table 3.

**Table 3:** Weighted mean difference of interventions versus placebo.

VS Placebo	Weighted mean difference CI 95%	Std. Err	Z	p
Lorazepam 0.5 mg	−3.720 [−7.435–0.005]	1.895	−1.96	0.050
Paroxetine 20 mg	−2.763 [−4.993–0.533]	1.137	−2.43	0.015
Silexan 160 mg	−4.963 [−7.167–2.759]	1.124	−4.41	<0.001
Silexan 80 mg	−3.820 [−5.261–2.380]	0.735	−5.20	<0.001

I^2^ = 0.00%, Tau^2^ = 0.

**Figure 5:**
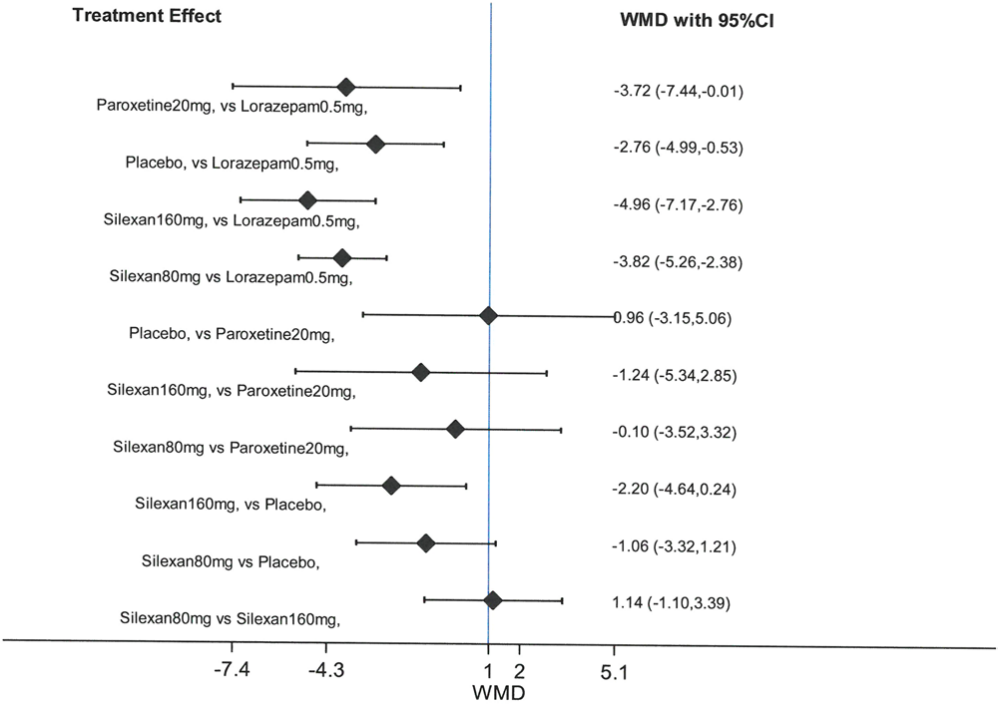
Pairwise comparison of all treatments.

Furthermore, a pairwise analysis was carried out to estimate the comparative effect of all the treatments versus placebo and other interventions. Using the pairwise comparison, a league table was generated to present all possible pairwise comparisons between any two of the five treatments (Fig. 5). Treatment effect, that is, the weighted mean difference of each pairwise comparison was calculated and reported along with the 95% CI. It was revealed that lorazepam 0.5 mg had no positive effect and did not cause a decline in HAMA score. The treatment with silexan 160 mg was found to be effective in comparison to placebo, paroxetine 20 mg, and lorazepam 0.5 mg. Overall, the effect of silexan 160 mg [−1.14 (−1.10, 3.39)], assessed on the basis of declining HAMA score, was superior in comparison to silexan 80 mg. In addition, the effect of silexan 80 mg [−1.06 (−3.32,1.21)] was observed to be slightly better than that of placebo. The decline in HAMA score was higher for silexan 160 mg in comparison to placebo and paroxetine 20 mg. Overall, all interventions including placebo were observed to be more effective in declining HAMA score compared to lorazepam 0.5 mg. Details are described in Table 4.

**Table 4:** League table for the effect of all interventions on HAMA scale.

Silexan 80 mg				
1.14 (−1.10,3.39)	Silexan 160 mg			
−1.06 (−3.32,1.21)	−2.20 (−4.64,0.24)	Placebo		
−0.10 (−3.52,3.32)	−1.24 (−5.34,2.85)	0.96 (−3.15,5.06)	Paroxetine 20 mg	
−3.82 (−5.26,−2.38)	−4.96 (−7.17, −2.76)	−2.76 (−4.99, −0.53)	−3.72 (−7.44, −0.01)	Lorazepam 0.5 mg

#### Safety of silexan

In the analyzed 5 studies, adverse events, attributable to silexan use, consisted mainly of gastrointestinal problems such as nausea, eructation or breath odour, and diarrhea. A number of patients also reported having headaches in the 2015 study by Kasper *et al*.^41^ Nevertheless, the number of patients that experienced these mild adverse events comprised of a small percentage of the sample size. No serious adverse event was found to be linked to the use of silexan.

Fatigue is a known side effect of lorazepam. In the study conducted by Woelk & Schlafke^38^, 16.2% of patients receiving treatment with lorazepam had fatigue whereas none in the silexan group experienced fatigue. However, 10.0% in the silexan group had nausea whereas only 2.7% had nausea in the lorazepam group.

The actual number and type of total adverse events experienced by the participants in the study conducted by Kasper *et al*.^36,37^ were not reported in details. Nonetheless, it was noted by Kasper *et al*.^37^ that the participants receiving treatment with silexan experienced a 3% increase in risk of gastrointestinal problems compared to those in the placebo group. Further details on adverse events reported in the studies can be found in Table 5.

**Table 5:** Side-effects reported among the participant consumed placebo and intervention treatment.

Author	Year	Intervention/Control	Type of Adverse Event (AE)								
			Nausea	Eructation/breath odour	Dyspepsia	Fatigue	Headache	Diarrhoea	Gastritis	Oral discomfort	Naso-pharyngitis
Woelk & Schlafke^35^	2009	80 mg/d Silexan	4 (10.0%)	3 (7.5%)	2 (5.0%)	—	—	—	—	—	—
		0.5 mg/d Lorazepam	1 (2.7%)	—	—	6 (16.2%)	—	—	—	—	—
Kasper *et al*.^38^	2015	80 mg/d Silexan	—	6 (7.0%)	—	—	—	1 (1.2%)	1 (1.2%)	1 (1.2%)	—
		Placebo	—	0	—	—	—	—	—	—	—
Kasper *et al*.^39^	2016	80 mg/d Silexan	6 (3.8%)	32 (20.0%)	—	—	5 (3.1%)	3 (1.9%)	—	—	3 (1.9%)
		Placebo	2 (1.3%)	0	—	—	11 (7.1%)	7 (4.5%)	—	—	8 (5.1%)
Kasper *et al*.^36^	2010	80 mg/d Silexan	—	—	—	—	—	—	—	—	—
		Placebo	—	—	—	—	—	—	—	—	—

## Discussion

To date, perhaps it is the first NMA estimating the effect of oral dosage form of lavender i.e. silexan in comparison to placebo, paroxetine and lorazepam on the anxiety score at the HAMA scale. Systematic reviews and meta-analysis have proven to be an effective tool to estimate the clinical efficacy and safety of herbs, and further comparative analysis using network meta-analysis will provide researcher an opportunity to estimate the clinical efficacy and safety of a herb versus various treatments or comparators^43^. Therefore, the current systematic review and NMA will serve as a useful tool in analyzing the use of silexan in clinical settings in an effective manner. The overall analysis revealed that administration of silexan 160 mg produced a higher decline in HAMA score [WMD −1.14 (−1.10, 3.39)] in comparison to silexan 80 mg. The effect of silexan 80 mg was observed to be similar to paroxetine 20 mg. Overall, the treatment with silexan 160 mg resulted in more significant decline in the HAMA score across all the comparators and therefore can be considered as the most effective anxiolytic intervention for the included studies. However, it should be noted that the dose of lorazepam used in the included RCT, and subsequently in this NMA, was only 0.5 mg which is the lowest daily dose recommended. Comparative efficacy of silexan with higher dose of lorazepam is not known and needs further research.

The lavender in different forms has been used in cosmetic and therapeutic applications for centuries. These applications were driven by unique scent of lavender essential oil. This essential oil became popular in aromatherapy and its therapeutic effectiveness in this form has been assessed clinically in a number of trials. Particularly, aromatherapy with lavender essential oil was effective in managing preoperative stress and anxiety in various settings significantly decreasing anxiety level compared to placebo^44–46^. However, similar interventions applied after surgery revealed no difference with control group on anxiety level and mental stress^47,48^.

The aromatherapy with lavender essential oil improved quality of sleep and reduced level of anxiety in patients with coronary artery disease^49^, significantly reduced anxiety in older patients with acute coronary syndrome^50^ and myocardial infarction^51^. Lower indexes for perceived stress and objective stress were observed in the intensive care unit patients receiving aromatherapy with lavender essential oil^52^. The inhaled lavender essential oil helped to alleviate anxiety in burn patients^53^ and postpartum women^54^.

However, no significant long-term improvements were observed in control of anxiety for patients suffering from cancer receiving aromatherapy treatment with lavender essential oil^55^. No significant difference in the anxiety level was also observed between cancer patients receiving inhalation with lavender essential oil during radiotherapy and the placebo group^56^. Overall, the aromatherapy with lavender as an anxiolytic agent has not been supported by sufficient evidence of therapeutic efficacy^57^. However, our NMA was based on the systemic administration of the lavender essential oil (Silexan) and demonstrated positive evidences for its anxiolytic effect. More detailed studies are required for the conclusion regarding other methods for the administration of lavender essential oil, particularly topically or via inhalation.

### Strengths and limitations

The exclusion of non-English articles can be one of the limitations of this NMA. This was mainly due to a lack of resources to translate articles written in other languages. There is a slight possibility that some relevant data from these non-English literature, if combined with the current analysis, might change the significance of lavender effects versus other comparators. In addition, all the studies investigating the effect of lavender used its essential oil in form of Silexan capsules. These studies were from Germany and the outcome might be potentially affected by the patients’ genetics. To get more comprehensive analysis and conclusions on the effect of lavender and particularly Silexan capsules, similar clinical studies using ethnically more diverse populations are required. The lavender preparations like Silexan capsules might have different results on HAMA scale in other races from different genetic makeup. Moreover, the current analysis estimated only the overall effect of the treatments on HAMA score, the current study was unable to compare the multiple treatments based on the decline in symptoms, severity and relapse of anxiety, which was mainly due to variable reporting of the results across the selected studied for the NMA.

Overall, the other bias was observed across all the five included studies. However, one of the main strength of this NMA is heterogeneity among the one-on-one comparison and pairwise comparison was 0% which reflects no inconsistencies among the studies. A number of factors including similarities between the study populations across RCTs included in this NMA as all studies were conducted in Germany, similarities in intervention (all in the form of Silexan capsules) and duration of follow-up and use of same tool (HAMA) to assess anxiety in all included studies could explain low heterogeneity observed in NMA.

## Conclusion

Results (through pairwise comparisons) revealed statistically significant effect of silexan 160 mg versus placebo, silexan 80 mg and paroxetine 20 mg. In addition, the effect of paroxetine 20 mg was also observed superior than placebo and silexan 80 mg. However, silexan consumption has shown some gastrointestinal side effect such as nausea, eructation or breath odor and diarrhea, which were tolerated by the patients recruited in the silexan arm. There is a need of more methodological strong studies to further investigate the effect of silexan among the patients from other regions to get a comprehensive picture about its clinical efficacy and safety.

## Data Availability

All materials, data and associated protocols are available to readers without restrictions.
